# Intravenous administration of human Muse cells recovers blood flow in a mouse model of hindlimb ischemia

**DOI:** 10.3389/fcvm.2022.981088

**Published:** 2022-11-11

**Authors:** Yusuke Hori, Tomoya Kitani, Kenji Yanishi, Takaomi Suga, Masaya Kogure, Tetsuro Kusaba, Yoshihiro Kushida, Mari Dezawa, Satoaki Matoba

**Affiliations:** ^1^Department of Cardiovascular Medicine, Graduate School of Medical Science, Kyoto Prefectural University of Medicine, Kyoto, Japan; ^2^Department of Nephrology, Graduate School of Medical Science, Kyoto Prefectural University of Medicine, Kyoto, Japan; ^3^Department of Stem Cell Biology and Histology, Tohoku University Graduate School of Medicine, Sendai, Japan

**Keywords:** peripheral arterial disease, hindlimb ischemia, cell therapy, Muse cell, mesenchymal stem cell

## Abstract

Cell-based therapies hold great promise for the treatment of peripheral arterial disease (PAD), especially in patients presenting with severe limb ischemia, although the optimal strategy remains to be explored. In this study, we evaluated the therapeutic effect of intravenous administration of human Muse cells, a unique subpopulation of mesenchymal stem cells (MSC), using a mouse model of hindlimb ischemia (HLI) without an immunosuppressant. Compared with the phosphate buffered saline (PBS) or non-Muse MSC groups, the Muse group showed significantly higher laser doppler blood flow in the ischemic limb at days 7 and 14 after HLI. Increased microvascular density [percent area of CD31(+) cells] and reduced interstitial fibrosis in the ischemic limb muscle were also observed in the Muse group. mCherry-expressing Muse cells were found in the ischemic border zone and expressed CD31 but did not in the non-ischemic limb. Muse cells produced higher amounts of vascular endothelial growth factor (VEGF) than non-Muse cells under normoxic and hypoxic conditions *in vitro*. In the ischemic muscle, tissue VEGF concentration and angiogenesis-related genes such as *Vegfa*, *Angpt1*, *Pdgfb*, and *Igf1* were significantly higher in the Muse group than in the other two groups. In addition, the proportion of M2 macrophages to total macrophages and the ratio of anti-inflammatory-related genes such as *IL-10*, *Arg1*, and *CD206 per iNOS* were significantly higher in the Muse group than in the other two groups. In summary, Muse cells exert pleiotropic effects in a mouse model of HLI, and therefore may provide a novel therapeutic approach for the treatment of PAD patients with severe limb ischemia.

## Graphical Abstract

Intravenously administered human-Muse cells homed to mouse ischemic limb and delivered pleiotropic effects, including differentiation into vascular cells, neovascularization, and anti-fibrosis/anti-inflammatory effects, leading to the successful recovery of blood flow without immunosuppressant. Muse cells may thus provide a novel approach for treating limb ischemia. 
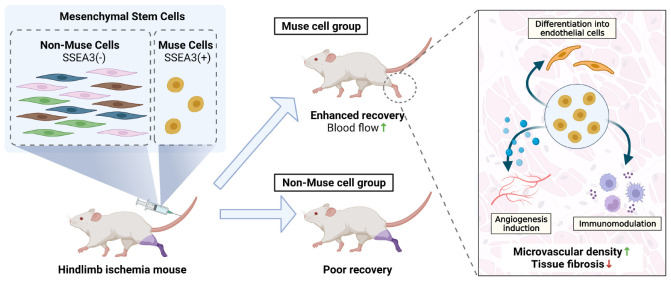

## Introduction

Peripheral artery disease (PAD) is a common circulatory disorder characterized by a narrowing of the peripheral arteries, most commonly caused by atherosclerosis. In the advanced stage, PAD patients often suffer from chronic pain, ulceration, and gangrene due to severe limb ischemia. Despite recent advances in treatment, a substantial number of advanced stage PAD patients, especially with hemodialysis, high frailty or low cardiac function, continue to suffer these symptoms and remain at high risk for subsequent amputations (1). The global burden of PAD is projected to rise over the next decade given the increasing prevalence of atherosclerotic diseases worldwide. Therefore, a considerable unmet therapeutic need remains for PAD patients.

Over the past decades, cell-based regeneration therapy has been extensively studied as a promising approach for treating PAD patients, and a number of studies have been conducted with several types of cells, including endothelial progenitor cells and bone marrow mononuclear cells, to validate their therapeutic effects on PAD (2). Several challenges must be overcome, however, before cell-based therapies can become a standard treatment option for PAD patients. These include the potential risk of teratoma formation, need for invasive procedures, high medical cost, and limited therapeutic efficacy (3, 4). Thus, further exploration is warranted to identify more suitable cell sources for cell-based treatments for ischemic limb.

Multilineage-differentiating stress-enduring (Muse) cells are pluripotent-like endogenous stem cells found as pluripotency surface marker stage-specific embryonic antigen (SSEA)-3-positive cells in the bone marrow, peripheral blood, and connective tissues of various organs; express pluripotency markers such as Oct3/4, NANOG, and SOX2; and exhibit stress tolerance by a high capacity for sensing and repairing DNA damage (5–7). The SSEA-3(+) Muse cells are collectable as several percent of cultured mesenchymal stem cells (MSCs) and fibroblasts (5, 8). Muse cells can differentiate spontaneously into triploblastic-lineage cells from a single cell and are self-renewable (5, 8).

Previous studies have demonstrated that systemically administered Muse cells preferentially home to damaged tissue in various animal models of disease including stroke, acute myocardial infarction, liver cirrhosis, chronic kidney injury, epidermolysis bullosa, and aortic aneurysm (9–15). Notably, systemically circulating Muse cells are considered to home to damaged tissues more efficiently than locally implanted Muse cells. Indeed, intravenous injection of Muse cells resulted in higher cell engraftment rather than local injection in a mouse model of amyotrophic lateral sclerosis (16). In addition, the safety and efficiency of intravenous injection of Muse cells without HLA matching or immunosuppression has been shown in patients with acute myocardial infarction and epidermolysis bullosa (17, 18).

Based on these findings, further clinical trials are currently underway in patients with stroke, acute myocardial infarction, epidermolysis bullosa, amyotrophic lateral sclerosis, spinal cord injury, perinatal hypoxic ischemic encephalopathy, and COVID-19-acute respiratory distress syndrome under the approval of regulatory authorities (Japan Pharmaceutical Information Center-Clinical Trials Information; JapicCTI-183834, 184103, 184563, and 194841). However, the therapeutic potential of the Muse cells in PAD remains unclear.

Here, we investigated the effect of intravenously administered human Muse cells in a mouse model of hindlimb ischemia (HLI) model without immunosuppressant treatment.

## Materials and methods

### Preparation of muse cells and non-muse cells

Human Muse cells and non-Muse MSCs were isolated as SSEA3-positive and -negative cells, respectively, from human bone marrow-derived mesenchymal stem cells (MSCs) (LONZA, PT2501) by fluorescence-activated cell sorting (FACS) (SONY, SSH800) with anti-SSEA3 antibody (Bio Legend, 330302), as described previously (6). The mCherry-expressing Muse cells or mCherry-expressing non-Muse MSCs were obtained by FACS from MSCs infected with the lentiviral vector carrying mCherry, as described previously (10). Human Muse cells, non-Muse MSCs, and MSCs were maintained at 37°C in α-minimum Essential Medium Eagle Modification (α-MEM) containing 10% fetal bovine serum, 1 ng/mL basic fibroblast growth factor (Miltenyi Biotec), 2 mM GlutaMAX (ThermoFisher Scientific), and 0.1 mg/mL kanamycin in an atmosphere containing 5% CO_2_ unless otherwise specified.

### Animal model of hindlimb ischemia and cell delivery

BALB/c male mice (12–14 weeks old) were anesthetized with isoflurane via facemask. After a skin incision in the left inguinal region, the left femoral artery and vein were isolated and ligated twice with 5-0 silk sutures just distal to the inguinal ligament, followed by complete resection of vessels between the two ties. The skin wound was closed in layers with 5-0 silk sutures. The mouse body temperature was maintained using a heating pad during the procedure. One day after the HLI surgery, animals received an injection of either 3 × 10^4^ human-Muse cells (Muse group), human non-Muse MSCs (non-Muse group), or phosphate buffered saline (PBS group) from the tail vein without immunosuppressant and were examined for 14 days unless otherwise specified. All animal studies were approved by the Animal Care and Use Committee of Kyoto Prefectural University of Medicine.

### Analysis of limb blood flow and ischemia scoring

Blood flow was measured using a laser Doppler blood flow analyzer (Omegazone, Omegawave) under isoflurane-induced anesthesia and expressed as the ratio of the ischemic to non-ischemic hindlimb. The mouse body temperature was maintained using a heating pad during the measurement. The degree of hindlimb ischemia was visually evaluated using the Modified Ischemia Score as reported previously (19).

### Immunohistochemical staining

Adductor muscles were harvested at day 5, day 7, or week 2 after hindlimb ischemia (HLI) surgery. The tissues were fixed for 1 h in 4% paraformaldehyde and incubated overnight in 30% sucrose solution. The tissues were then embedded in OCT compound (Sakura Finetek), snap-frozen in liquid nitrogen, and sectioned on a cryostat. For the antibody reaction, tissue sections were incubated overnight with rabbit anti-cluster of differentiation 31 (CD31, Abcam, ab182981) at a 1:5,000 dilution, rat anti-macrophage/monocyte (MOMA2, Gene Tex, GTX39773) at a 1:50 dilution, or rabbit anti-arginase-1 (Arg1, Cell Signaling, 93668) at a 1:100 dilution. Goat anti-rabbit Alexa 488, goat anti-rabbit Alexa 594, or goat anti-rat Alexa 488 (Abcam) was used as secondary antibodies. After mounting with Prolong GOLD Antifade with 4′,6-diamidino-2-phenylindole (DAPI, Thermo Fisher Scientific), imaging was performed using BZ-X800 Light Microscope (Keyence). For phalloidin staining, tissue sections were incubated with Phalloidin-FITC (Sigma-Aldrich) at a 1:100 dilution for 1 h. CD31 was also visualized using a standard HRP-DAB (horseradish peroxidase-3,3′-diaminobenzidine) staining with rabbit anti-CD31 (Abcam, ab182981) at a 1:1,000 dilution.

### Quantification of microvascular density, interstitial fibrotic area, and immune cell population

Frozen tissue sections were stained with CD31, Masson’s trichrome, or MAMA2 and Arg1 for evaluating the microvascular density, interstitial fibrotic area, or immune cell population, respectively.

The stained tissue sections were imaged at 20× magnification using BZ-X800 Light Microscope (Keyence). CD31-positive area, Trichrome-positive area, MOMA2-positive area, or MOMA2/Arg1-double positive area were calculated using BZ-X800 Analyzer Software (Keyence) in five randomly selected fields in each mouse, respectively.

### Flow cytometric analysis of engrafted cells

Human mCherry-expressing Muse cells (3 × 10^4^), non-Muse MSCs (3 × 10^4^) were intravenously administered 1 day after HLI surgery. PBS was injected as a control. At 7 days after HLI surgery, the ischemic and non-ischemic adductor muscles were harvested and enzymatically and mechanically dissociated into single cell suspension using Skeletal Muscle Dissociation Kit (Miltenyi Biotec, 130-098-305) with gentleMACS Dissociator (Miltenyi Biotec) according to the manufacturer’s instructions. After erythrocytes were lysed using BD Pharm Lyse (BD Biosciences, 555899), the cells were subjected to flow cytometric analysis (SONY, MA900). For cell surface marker analysis, the cells were stained with FITC-conjugated anti-human/mouse CD31 antibody (Invitrogen, 11-0311-81) at a 1:100 dilution or FITC-conjugated anti-human NG2 antibody (Miltenyi Biotec, 130-098-794) at a 1:20 dilution before the flow cytometric analysis. FITC-conjugated rat IgG2a (Biolegend, 400505) or FITC-conjugated mouse IgG1 (Biolegend, 400107) were used as isotype control, respectively. For analysis of smooth muscle myosin heavy chain (SM-MHC), the cells were fixed and permeabilized with PFA and methanol, and stained with mouse anti-human SM-MHC antibody (Dako, M3558) at a 1:100 dilution, followed by secondary antibody reaction with Alexa Fluor 647 conjugated anti-mouse IgG (Invitrogen, A21237) at a 1:1,000 dilution before the flow cytometric analysis. Mouse IgG1 (Biolegend, 401401) was used as isotype control. A total of 1,000 or 3,000 events were acquired in each unstained or stained sample, respectively.

### Detection of human genomic DNA

Human Muse cells (3 × 10^4^) were intravenously administered 1 day after HLI surgery. At 7 days after HLI surgery, genomic DNA was extracted from the mouse tissue using NucleoSpin Tissue (Macherey-Nagel) according to the manufacturer’s instructions. As previously reported (20), probe-based real-time polymerase chain reaction (PCR) was performed on a CFX 384 Real-Time system (Bio-Rad) using Probe qPCR Mix (Takara) with the following primers and probes: human *Alu* forward primer, 5′-CATGGTGAAAC CCCGTCTCTA-3′; human *Alu* reverse primer, 5′-GCCTCAGC CTCCCGAGTAG-3′; human *Alu* probe, 5′-FAM-ATTAGC CGGGCGTGGTGGCG-TAMRA-3′; mouse Y chromosome forward primer, 5′-TTTTGCCTCCCATAGTAGTATTTC CT-3′; mouse Y chromosome reverse primer, 5′-TGTAC CGCTCTGCCAACCA-3′; mouse Y chromosome probe, 5′-FAM-AGGGATGCCCACCTCGCCAGA-TAMRA-3′.

### Quantitative real-time PCR analysis of mRNA expression levels

Total RNA was extracted from the adductor muscle in the ischemic hindlimb using a Direct-zol RNA Kit (Zymo Research) and cDNA was synthesized using PrimeScript RT reagent Kit (Takara). Real-time PCR was performed on a CFX 384 Real-Time system using the KAPA SYBR FAST qPCR kit. The following primers were used: mouse *Gapdh* forward primer, 5′-AGGTCGGTGTGAACGGATTTG-3′; mouse *Gapdh* reverse primer, 5′-TGTAGACCATGTAGTTGAGGTCA-3′; mouse *Vegfa* forward primer, 5′-GCACATAGAGAGAATGAGC TTCC-3′; mouse *Vegfa* reverse primer, 5′- CTCCGCTCTGAAC AAGGCT-3′; mouse *Fgf2* forward primer, 5′- GCGACCC ACACGTCAAACTA-3′; mouse *Fgf2* reverse primer, 5′- CCGTCCATCTTCCTTCATAGC-3′; mouse *Angpt1* forward primer, 5′- ATCCCGACTTGAAATACAACTGC-3′; mouse *Angpt1* reverse primer, 5′- CTGGATGATGAATGTCTGAC GAG-3′; mouse *Pdgfb* forward primer, 5′- TGCTGCAC AGAGACTCCGTA-3′; mouse *Pdgfb* reverse primer, 5′- GATGAGCTTTCCAACTCGACTC-3′; mouse *Igf1* forward primer, 5′- CACATCATGTCGTCTTCACACC-3′; mouse *Igf1* reverse primer, 5′- GGAAGCAACACTCATCCACAATG-3′; mouse *iNOS* forward primer, 5′- GTTCTCAGCCCAACAAT ACAAGA-3′; mouse *iNOS* reverse primer, 5′- GTGGACG GGTCGATGTCAC-3′; mouse *IL10* forward primer, 5′- GCTCTTACTGACTGGCATGAG-3′; mouse *IL10* reverse primer, 5′- CGCAGCTCTAGGAGCATGTG-3′; mouse *Arg1* forward primer, 5′- CTCCAAGCCAAAGTCCTTAGAG-3′; mouse *Arg1* reverse primer, 5′- AGGAGCTGTCATTAGGGAC ATC-3′. All data were processed using the ΔΔCT method.

### Measurement of vascular endothelial growth factor concentration

For measurement of the vascular endothelial growth factor (VEGF) concentration in the cell culture supernatant, Muse cells or non-Muse MSCs were seeded on 48-well tissue culture plates at a density of 6.0 × 10^4^ cells/cm^2^ and allowed to grow for 3 days. On the day of the assay, the cells were washed with phosphate buffered saline and incubated in serum-free α-MEM under normoxic or hypoxic (0.1% O_2_) conditions overnight using the BIONIX hypoxic culture kit (Sugiyamagen), and then the cell culture supernatant was collected. For measurement of the tissue VEGF level, 3 × 10^4^ human Muse cells or non-Muse MSCs were intravenously administered 1 day after HLI surgery. At 3 days after HLI surgery, total protein was extracted from the adductor muscles using RIPA lysis buffer with protease inhibitor (Nacalai Tesque) and total protein concentrations were measured using the DC Protein Assay Kit (Bio-Rad). VEGF concentrations were measured using a Human VEGF ELISA Kit (KE00085, Proteintech) for the cell culture supernatant or a Mouse VEGF ELISA Kit (ab209882, Abcam) for the mouse tissue with an iMark Microplate reader (Bio-Rad) according to the manufacturer’s instructions.

### Statistical analyses

All data are expressed as mean ± SEM. Statistical comparisons were performed using GraphPad Prism version 9. Variables were tested for normal distribution using the Shapiro-Wilk test. Normally distributed data were analyzed by 1-way ANOVA followed by Holm-Sidak multiple comparison of more than two groups and Student’s t test for comparing two groups. Data that were not normally distributed were analyzed using Kruskal-Wallis test with Dunn’s multiple comparison for more than two groups and the Mann-Whitney *U* test for two groups.

## Results

**FIGURE 1:**
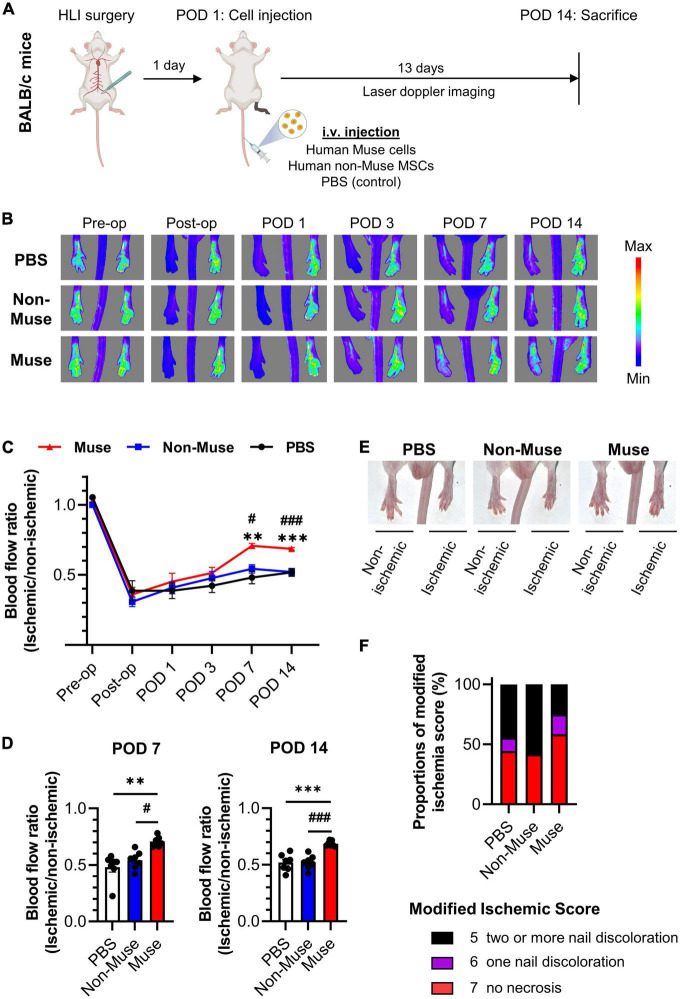
Muse cell intravenous injection recovered blood flow in a mouse model of hindlimb ischemia. **(A)** Schematic outline of study workflow. Human-Muse cells, human-non-Muse mesenchymal stem cells (MSCs), or phosphate buffered saline (PBS, control) were administered via intravenous injection (i.v.) on postoperative day (POD) 1 after hindlimb ischemia (HLI) surgery. **(B–D)** Representative Laser Doppler blood perfusion images **(B)** and quantitative analysis of the ischemic/non-ischemic limb blood flow ratio over time **(C,D)**. The hindlimb blood flow ratio was measured before and after HLI surgery and on POD 1, 3, 7, and 14 (*n* = 7 per each group; mean ± SEM). Dots correspond to each mouse. ^**^P < 0.01 PBS vs. Muse; ^***^P < 0.001 PBS vs. Muse; ^#^*P* < 0.05 non-Muse vs. Muse;^###^*P* < 0.001 non-Muse vs. Muse. **(E,F)** Representative images **(E)** and scores **(F)** of necrotic changes in hindlimb at POD 14 (*n* = 9 in the PBS group, *n* = 11 in the non-Muse group, and *n* = 12 in the Muse group).

The effects of Muse cell administration on blood flow in the ischemic limb were evaluated by laser Doppler blood flow analysis at pre-operation (Pre-op), immediately after operation (Post-op), and up to 14 days after the operation (Figure 1A). No significant difference in the blood flow was detected among the three groups up to postoperative day (POD) 3. At POD 7, however, the Muse group exhibited significant improvement in the ischemic limb blood flow compared with the PBS (*P* < 0.01) and non-Muse groups (*P* < 0.05); no significant difference was detected between the PBS and non-Muse groups (Figures 1B–D). Recovery in the Muse group became more impressive at POD 14 compared with that in the PBS (*P* < 0.001) and non-Muse (*P* < 0.01) groups; no significant difference was detected between the PBS and non-Muse groups (*n* = 7 per each group) (Figures 1B–D). The Muse group also developed milder necrotic changes in the ischemic limb compared with the PBS and non-Muse groups at POD 14 (Figures 1E,F).

**FIGURE 2:**
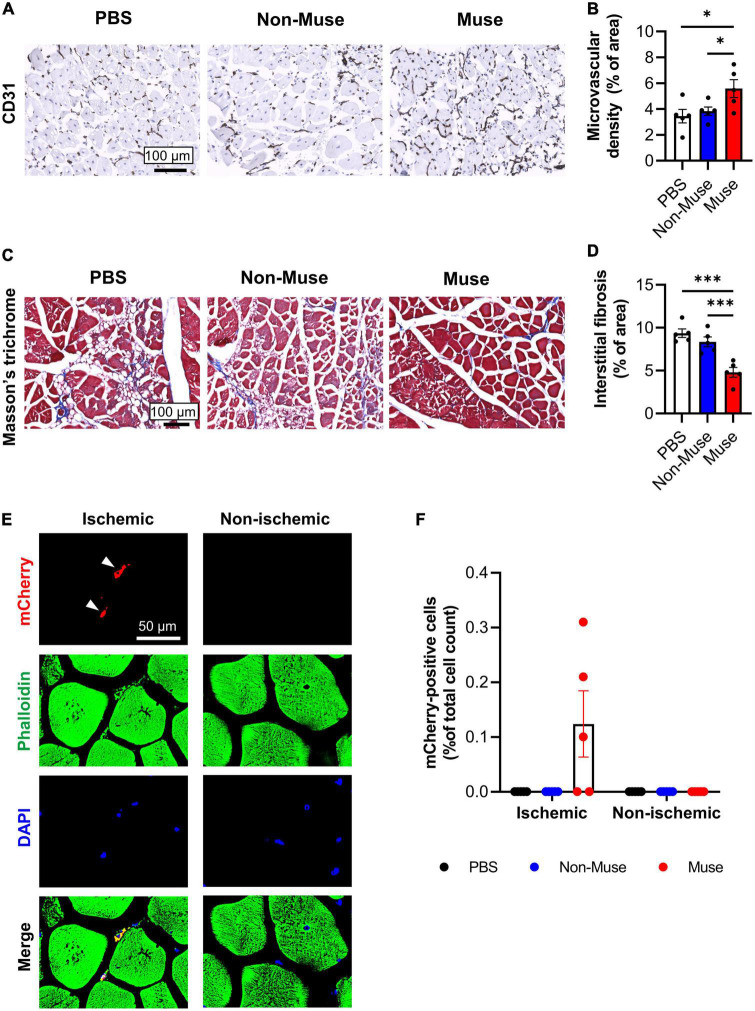
Muse cells enhanced angiogenesis and ameliorated muscle fibrosis in ischemic hindlimb. **(A)** Representative images of microvascular density in ischemic adductor muscles at 14 days after hindlimb ischemia (HLI) surgery. Tissue sections were stained with anti-CD31 antibody and detected with DAB (brown). Scale bar, 100 μm. **(B)** Quantitative analysis of the CD31-positive vessel area. Results are expressed as mean ± SEM (*n* = 5). Dots correspond to each mouse. *P < 0.05. **(C)** Representative images showing muscle fibrosis in the ischemic adductor muscles at 14 days after HLI surgery. Tissue sections were stained with Masson’s trichrome (blue = collagen fibers; red = muscle). Scale bar, 100 μm. **(D)** Quantitative analysis of the fibrotic area in Masson’s trichrome-stained ischemic adductor muscles. Results are expressed as mean ± SEM (*n* = 5). Dots correspond to each mouse. ^***^P < 0.001. **(E)** Representative images of ischemic and non-ischemic adductor muscle at 7 days after HLI surgery. Muse cells expressing mCherry were intravenously administered 1 day after HLI surgery. Tissue sections were stained with phalloidin (green) and DAPI (blue). White arrowheads indicate mCherry-expressing Muse cells in the ischemic border zone of the ischemic adductor muscle. Scale bar, 50 μm. **(F)** Detection of Muse cells in the adductor muscles by flow cytometry at 7 days after HLI surgery. Human mCherry-expressing Muse cells, non-Muse MSCs were intravenously administered 1 day after HLI surgery and PBS was injected as a control (*n* = 5 per each group). Results are expressed as mean ± SEM of the percentage of mCherry-positive cells per total cell count in the dissociated ischemic adductor muscle. Dots correspond to each mouse.

Histologic analysis was conducted in the ischemic limb at POD 14. Microvascular density as measured by the percent area of CD31-positive (vascular endothelial cell marker) cells in the ischemic adductor muscle was significantly increased in the Muse group compared to that in the PBS and non-Muse groups (both *P* < 0.05), while there was no significant difference between the PBS and non-Muse groups (*n* = 5 per each group) (Figures 2A,B and Supplementary Figures 1A–D, 2A). Interstitial fibrosis area in the ischemic adductor muscle was measured by Masson’s trichrome staining at POD 14. The fibrotic area was significantly smaller in the Muse group compared with the PBS and non-Muse groups (both *P* < 0.01), while there was no significant difference between the PBS and non-Muse groups (*n* = 5 per each group) (Figures 2C,D).

The engraftment of intravenously administered Muse cells in the ischemic adductor muscle was assessed by injection of 3 × 10^4^ mCherry-expressing Muse cells from the tail vein 1 day after HLI surgery. At POD 7, mCherry-expressing Muse cells were observed in the ischemic border zone of the adductor muscle, but not in the contralateral (non-ischemic) adductor muscle (Figure 2E). Flow cytometric analysis also revealed existence of mCherry-positive cells only in the ischemic adductor muscle after the injection of mCherry-expressing Muse cells at POD 7 (*n* = 5 per each group) (Figure 2F). In addition, the amount of human genomic DNA was examined in these mice by species-specific quantitative-polymerase chain reaction (qPCR) at POD 7. Human genomic DNA was detected in the ischemic limb, as well as in the lung and spleen, but not in the non-ischemic limb and tail (*n* = 3) (Supplementary Figure 3A).

**FIGURE 3:**
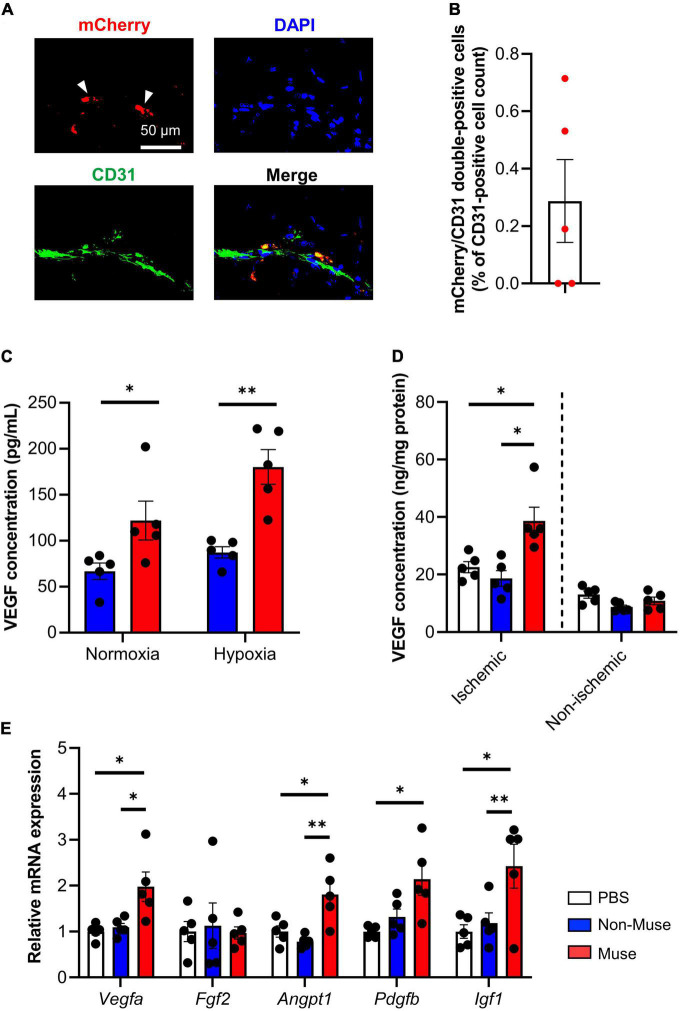
Angiogenic potential of muse cells. **(A)** Representative images showing vascular endothelial markers CD31 expression of mCherry-expressing Muse cells in the ischemic adductor muscles at 7 days after hindlimb ischemia (HLI) surgery. Tissue sections were stained with anti-CD31 antibody (green) and 4′6-diamidino-2-phenylindole (DAPI) (blue). Scale bar, 50 μm. **(B)** Flow cytometric analysis of CD31 expression in the ischemic adductor muscles at 7 days after HLI surgery (*n* = 5). Results are expressed as mean ± SEM of the percentage of mCherry/CD31 double-positive cells per total CD31-positive cell count in the dissociated ischemic adductor muscle. Dots correspond to each mouse. **(C)** Vascular endothelial growth factor (VEGF) concentration measured by enzyme-linked immunosorbent assay (ELISA) in the cell culture supernatant of Muse cells or non-Muse mesenchymal stem cells (MSCs) under normoxic and hypoxic conditions. Results are expressed as mean ± SEM (*n* = 5). Dots correspond to each biological replicate. **(D,E)** VEGF concentration measured by ELISA in the adductor muscles **(D)** and relative mRNA expression of representative angiogenic factors in the ischemic adductor muscle **(E)** at 7 days after HLI surgery. Muse cells or non-Muse MSCs were intravenously injected 1 day after HLI surgery and phosphate buffered saline was injected as a control. Results are expressed as mean ± SEM (*n* = 5). Dots correspond to each mouse. *P < 0.05; ^**^P < 0.01.

Next, *in vivo* differentiation potential of Muse cells in the ischemic limb was examined. Immunohistochemistry of the ischemic adductor muscle revealed that mCherry-expressing Muse cells expressed CD31 at POD 7 (Figure 3A and Supplementary Figure 2B). Flow cytometric analysis showed that ≈0.29% of total CD31-positive cells in the dissociated adductor muscle were double-positive for CD31 and mCherry (Figure 3B). On the other hand, human neuron-glial antigen 2 (NG2, a pericyte marker) and smooth muscle myosin heavy chain (SM-MHC, a vascular smooth muscle cell maker) were virtually under the detection limit in the dissociated ischemic adductor muscle (*n* = 5 per each group) (Supplementary Figures 3B,C).

To further understand the mechanisms by which Muse cell administration increased microvascular density in the ischemic adductor muscle, the angiogenic paracrine potential of Muse cells was evaluated *in vitro*. The concentration of human vascular endothelial growth factor (VEGF) in the cell culture supernatant of Muse cells or non-Muse MSCs was significantly higher in the Muse cell culture than in the non-Muse MSC culture under normoxic conditions (*P* < 0.05), and even higher under hypoxic conditions (0.1% O2) (*P* < 0.01) (*n* = 5 per each group) (Figure 3C). In addition, the mouse tissue VEGF concentration in the ischemic adductor muscle was significantly higher in the Muse group compared with the non-Muse group at POD 3 (*P* < 0.001) (*n* = 5 per each group) (Figure 3D). Quantitative PCR of the ischemic adductor muscle showed a higher expression level of angiogenesis-related genes such as *VEGF a* (*Vegfa*), *angiopoietin 1* (*Angpt 1*), *platelet-derived growth factor subunit b precursor* (*Pdgfb*), and *insulin-like growth factor 1* (*Igf1*) in the Muse group compared with the non-Muse group, while *fibroblast growth factor 2* (*fgf2*) did not differ among the 3 groups at POD 3 (*n* = 5 per each group) (Figure 3E).

**FIGURE 4:**
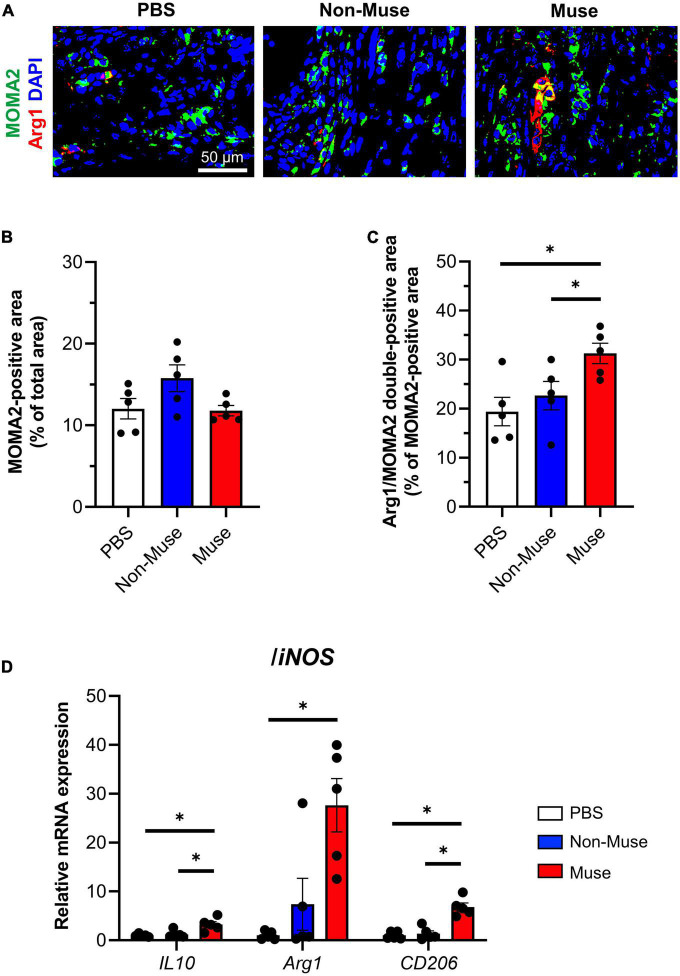
Muse cells modulated immune responses in ischemic hindlimb. **(A)** Representative immunofluorescence images of the macrophage/monocyte marker anti-macrophage/monocyte (MOMA2) (green) and the M2 macrophage marker arginase 1 (Arg1) (red) in the ischemic adductor muscle at 5 days after hindlimb ischemia (HLI) surgery. Cell nuclei were stained with 4′6-diamidino-2-phenylindole (DAPI) (blue). Scale bar, 50 μm. Muse cells or non-Muse mesenchymal stem cells (MSCs) were intravenously injected 1 day after HLI surgery and phosphate buffered saline (PBS) was injected as a control. **(B,C)** Quantification of MOMA2-positive area among total section area **(B)** and Arg1/MOMA2 double-positive area among total MOMA2-positive area **(C)**. Results are expressed as mean ± SEM (*n* = 5). Dots correspond to each biological replicate. **(D)** mRNA expression of M2 macrophage marker/anti-inflammatory genes relative to M1 macrophage marker/proinflammatory gene, *inducible nitric oxide synthase* (*iNOS*) in the ischemic adductor muscle at 7 days after HLI surgery. Muse cells or non-Muse MSCs were intravenously injected 1 day after HLI surgery and PBS was injected as a control. Results are expressed as mean ± SEM (*n* = 5). Dots correspond to each mouse. *P < 0.05.

The immune-inflammatory response in the ischemic adductor muscle, represented by inflammatory cell infiltration and macrophage polarization, was investigated. Double-staining of the monocyte/macrophage marker MOMA2 and M2 macrophage marker arginase1 (Arg1) revealed no significant difference in the total monocyte/macrophage infiltration among the 3 groups, while the percent of M2 macrophages was significantly higher in the Muse group compared with the other 2 groups (both *P* < 0.05) at POD 5 (*n* = 5 per each group) (Figures 4A–C and Supplementary Figure 4).

The expression of inflammation-related genes in the ischemic adductor muscle based on qPCR was investigated at POD 7. The ratios of anti-inflammatory genes, such as *interleukin-10*, *arginase1*, and *CD206*, to proinflammatory gene *inducible nitric oxide synthase* (*iNOS*) were all higher in the Muse group than in the other 2 groups (all *P* < 0.05), except for the *arginase1*/*iNOS* ratio between the Muse and non-Muse groups (*n* = 5 per each group) (Figure 4D).

## Discussion

Over the past few decades, accumulating evidence has revealed that cell-based therapy using bone marrow-derived cell populations, including mononuclear cells, endothelial precursor cell, and MSCs, has low safety concerns, but limited therapeutic effects on PAD patients with severe limb ischemia. Therefore, cell sources with high safety and high therapeutic effects have been explored (4, 21).

In the present study, we demonstrated that intravenous administration of Muse cells increased the microvascular density in ischemic limbs and recovered blood flow. We found engrafted Muse cells expressing vascular endothelial marker CD31 in the ischemic limb, suggesting the *in vivo* differentiation potential of Muse cells into vascular endothelial cells may contribute to vascular regeneration in the ischemic limb. VEGF secretion from Muse cells was higher than that from non-Muse MSCs and mouse tissue VEGF levels in the ischemic limb were higher in the Muse group than in the non-Muse group. Consequently, the greater angiogenic factor production and vascular differentiation potential of Muse cells may contribute to blood flow recovery in the ischemic limb. In addition, Muse cell administration restrained tissue fibrosis, partly due to the suppression of inflammation, as represented by the increase in anti-inflammatory factors such as *interleukin-10*, *arginase1*, and *CD206* rather than an increase of proinflammatory *iNOS* in the ischemic limb. Because tissue fibrosis and inflammation are tightly intertwined, inflammatory cell infiltration and macrophage polarization were examined in the ischemic limbs. In the Muse cell group, M2 macrophages, which play a key role in resolution of inflammation (22), were increased. Muse cells are reported to achieve tissue repair partly through differentiation into vascular cells (9, 13) and anti-fibrotic/anti-inflammatory mechanisms via immunomodulatory effects (7, 23). Consistent with these previous findings, our results suggest that Muse cells restored blood flow and inhibited excessive fibrosis in ischemic limbs via pleiotropic effects including vascular cell differentiation, production of angiogenic factors, and immunomodulation.

It is noteworthy that human Muse cells exhibited therapeutic potential in a mouse model of HLI without immunosuppression. Although there is still considerable debate regarding immune rejection of xenogeneic cell transplantation, numerous studies have reported the therapeutic effects of human MSCs in mice owing to their immunomodulatory effects (24). Muse cells express HLA-G, which is associated with immune tolerance in the placenta, while fewer than 20% of adult human MSCs express HLA-G (9, 25, 26). Similar to previous reports showing that intravenously administered xenogeneic (human) Muse cells escaped from immune rejection and survived in the host tissue as differentiated tissue-comprising cells in animal models of adriamycin-induced nephropathy (mouse), lung ischemia-reperfusion injury (rat), myocardial infarction (rabbit), and amyotrophic lateral sclerosis (mouse) (9, 10, 16, 27), engraftment of intravenously injected human Muse cells also confirmed in the ischemic limb tissue in the mouse model of HLI. Indeed, clinical trials have shown the safety and efficiency of intravenous administration of Muse cells without HLA-matching or immunosuppressant treatment (17, 18). Additionally, we found that Muse cell administration increased endogenous angiogenic factors in the ischemic limb, suggesting the contribution of indirect effects of Muse cells to angiogenesis. Supporting this idea, we also found that Muse cells administration increased anti-inflammatory M2 macrophages, which are also known to promote angiogenic processes, in the ischemic limb. Consistent with this finding, a previous study demonstrated that conditioned medium of human Muse cells reduced LPS-stimulated production of proinflammatory cytokine in mouse macrophage-like RAW cells and mouse peritoneal macrophages (23). These results imply that immunomodulatory effects of Muse cells also play a significant role in the enhanced angiogenic response in the ischemic limb.

In recent years, intravenous administration of human MSCs has been intensively studied as a new therapeutic approach for various diseases; however, its therapeutic efficacy is needed to be improved and injection of a large amount of MSCs carries a risk of pulmonary embolism (28). Muse cell is a subpopulation of MSCs with high therapeutic potential, comprising several percent of human bone marrow-derived MSCs (5, 8). In our study, ≈5% of total MSCs were isolated as Muse cells after *in vitro* expansion of MSCs to be used in the following experiments (Supplementary Figures 5A,B). Importantly, our data demonstrated that Muse cells exerted a therapeutic effect in the HLI mouse model, while non-Muse MSCs did not, implying that therapeutic effect of MSCs on limb ischemia in previous studies might be primary derived from a small amount of Muse cells contained in MSCs (29). In line with the previous studies demonstrating that Muse cells have higher tissue repair capacity compared with non-Muse MSCs (9, 13, 30), our result suggest that Muse cell therapy has potential to provide safer and more effective cell therapy compared to conventional MSC therapy (6).

A potential major safety concern of angiogenesis therapies for ischemic diseases has been angiogenesis in non-targeted tissues. Muse cells produced relatively high amounts of VEGF even under non-hypoxic condition and intravenously administered Muse cells were detected in non-target tissues such as the lung and the spleen in our study, assuming the possible aberrant angiogenesis in these tissues. Interestingly, however, Yamada et al. previously demonstrated that the majority of Muse cells engrafted into non-injured tissues such as the lung disappear by 2 weeks after intravenous injection, probably because Muse cells had no chances to be incorporated into the tissue by replacing damaged cells in non-injured tissues (9). In addition, angiogenetic factors including VEGF are considered not to induce substantial angiogenesis unless tissues are exposed to ischemic conditions (31). Furthermore, side effects related to an aberrant angiogenesis have not been reported in human clinical trials using intravenous injection of Muse cells (17, 18). Therefore, angiogenesis in non-targeted tissues is expected to be less frequent in patients receiving Muse cells intravenously, although careful attention should be paid to the possible aberrant angiogenesis in future clinical trials.

All taken together with previous studies, Muse cell-based therapy holds several potential advantages for regenerative treatments of limb ischemia. First, Muse cells can be administered via intravenous injection, which is a simple and less invasive delivery route for cell therapy. Second, allogenic Muse cell transplantation is therapeutically effective without HLA-matching or immunosuppressant treatment, which allows off-the-shelf cell therapy to provide a timely and cost-effective treatment for the patients. Third, based on the previous two advantages, Muse cell therapy can be repeatedly performed in the same patient, which might lead to better patient outcomes compared to other cell therapies for the treatment of limb ischemia.

In summary, intravenous administration of Muse cells recovered blood flow and reduced tissue fibrosis in ischemic limbs in a mouse model of HLI. The therapeutic effects of Muse cells may be attributed to their pleiotropic effects, including vascular cell differentiation, production of angiogenic factors, and anti-inflammation and anti-fibrosis effects. Our study is the first to demonstrate that Muse cell-based therapy could be an attractive therapeutic option for PAD patients with severe limb ischemia.

## Data availability statement

The raw data supporting the conclusions of this article will be made available by the authors, without undue reservation.

## Ethics statement

This animal study was reviewed and approved by the Animal Care and Use Committee of Kyoto Prefectural University of Medicine.

## Author contributions

YH and TS: collection and/or assembly of data. TKi: conception and design, data analysis and interpretation, and manuscript writing. KY: conception and design. MK and YK: support of cell culture and preparation of mCherry-Muse cells. TKu: support for immunohistochemistry. MD: manuscript writing. SM: conception and design, financial support, administrative support, and final approval of manuscript. All authors contributed to the article and approved the submitted version.
