# Supplementary figures and images for: Intravenous administration of human Muse cells recovers blood flow in a mouse model of hindlimb ischemia
Source: Front Cardiovasc Med. 2022 Nov 11;9:981088. doi: 10.3389/fcvm.2022.981088 (PMC9692087; doi:10.3389/fcvm.2022.981088)

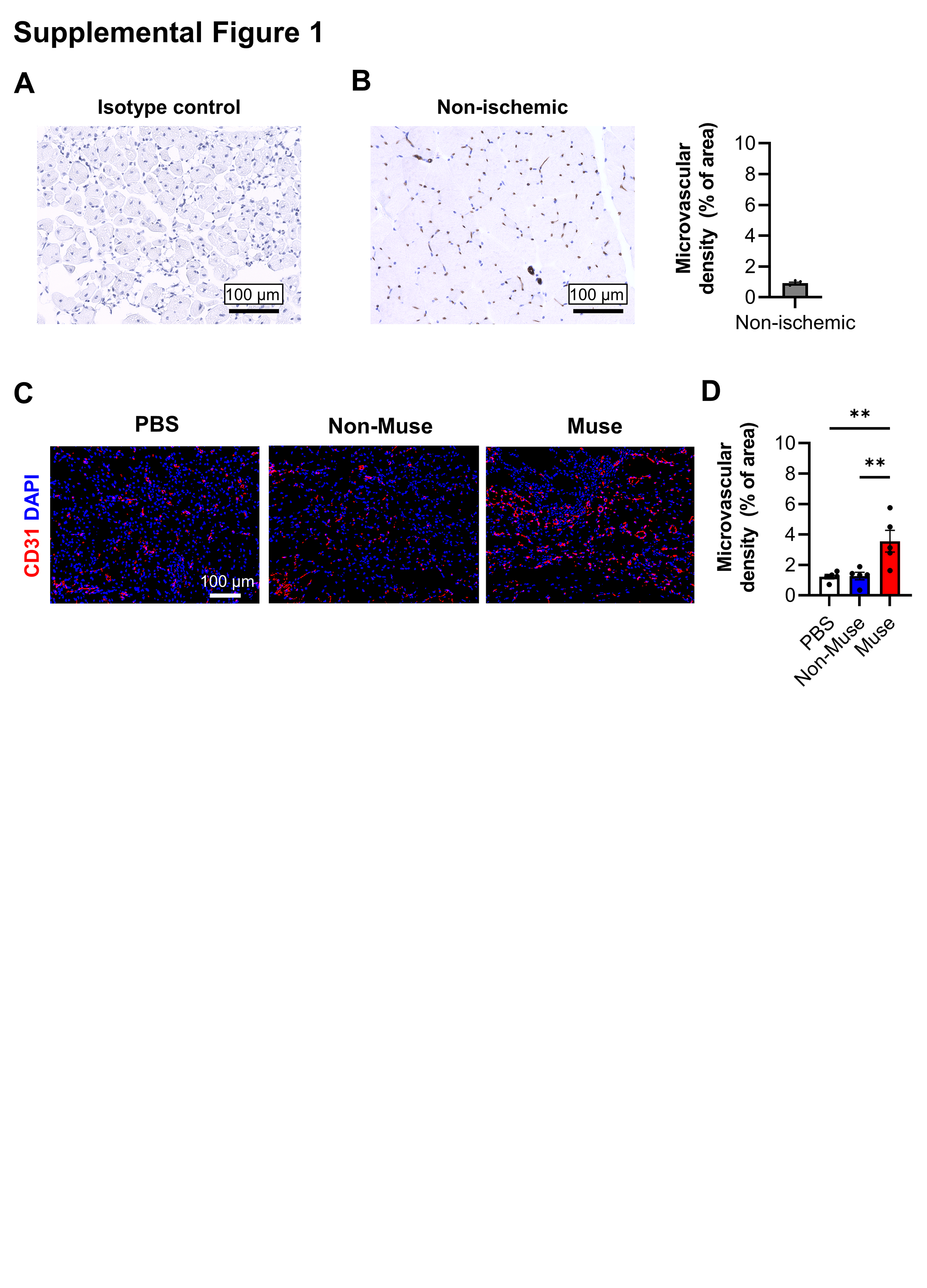

Supplement: Supplementary file 1 [file Image_1.TIF]

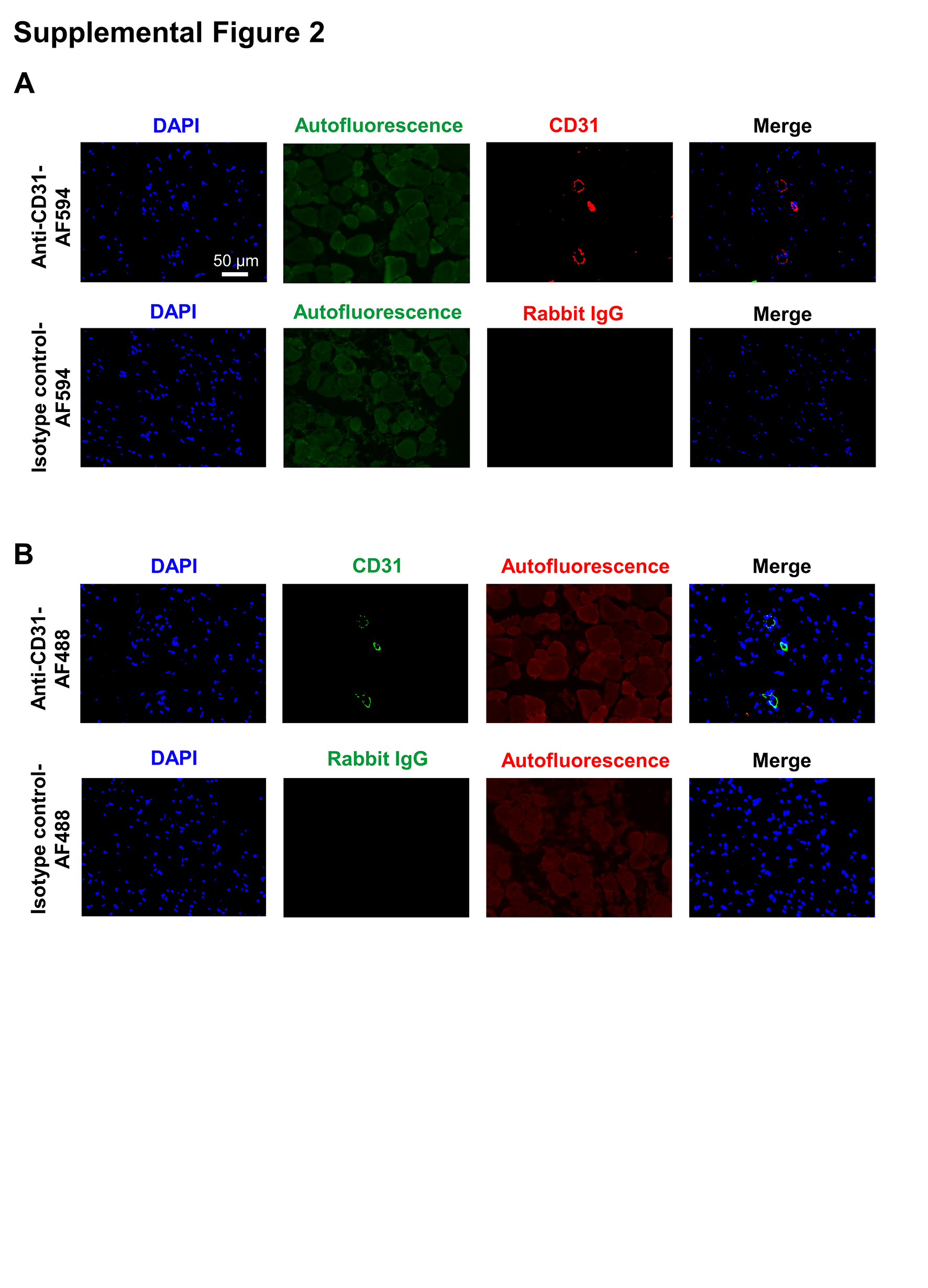

Supplement: Supplementary file 2 [file Image_2.TIF]

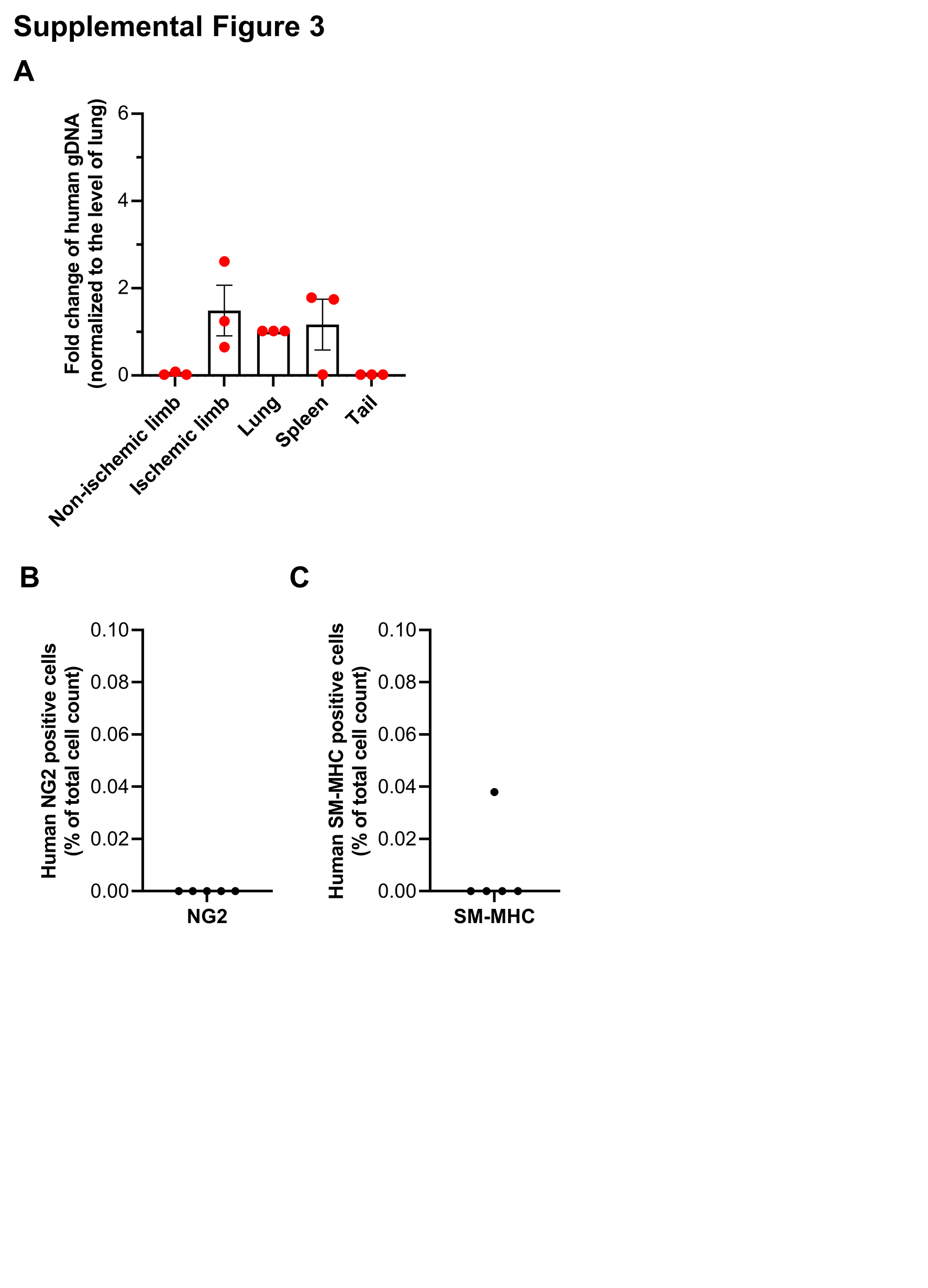

Supplement: Supplementary file 3 [file Image_3.TIF]

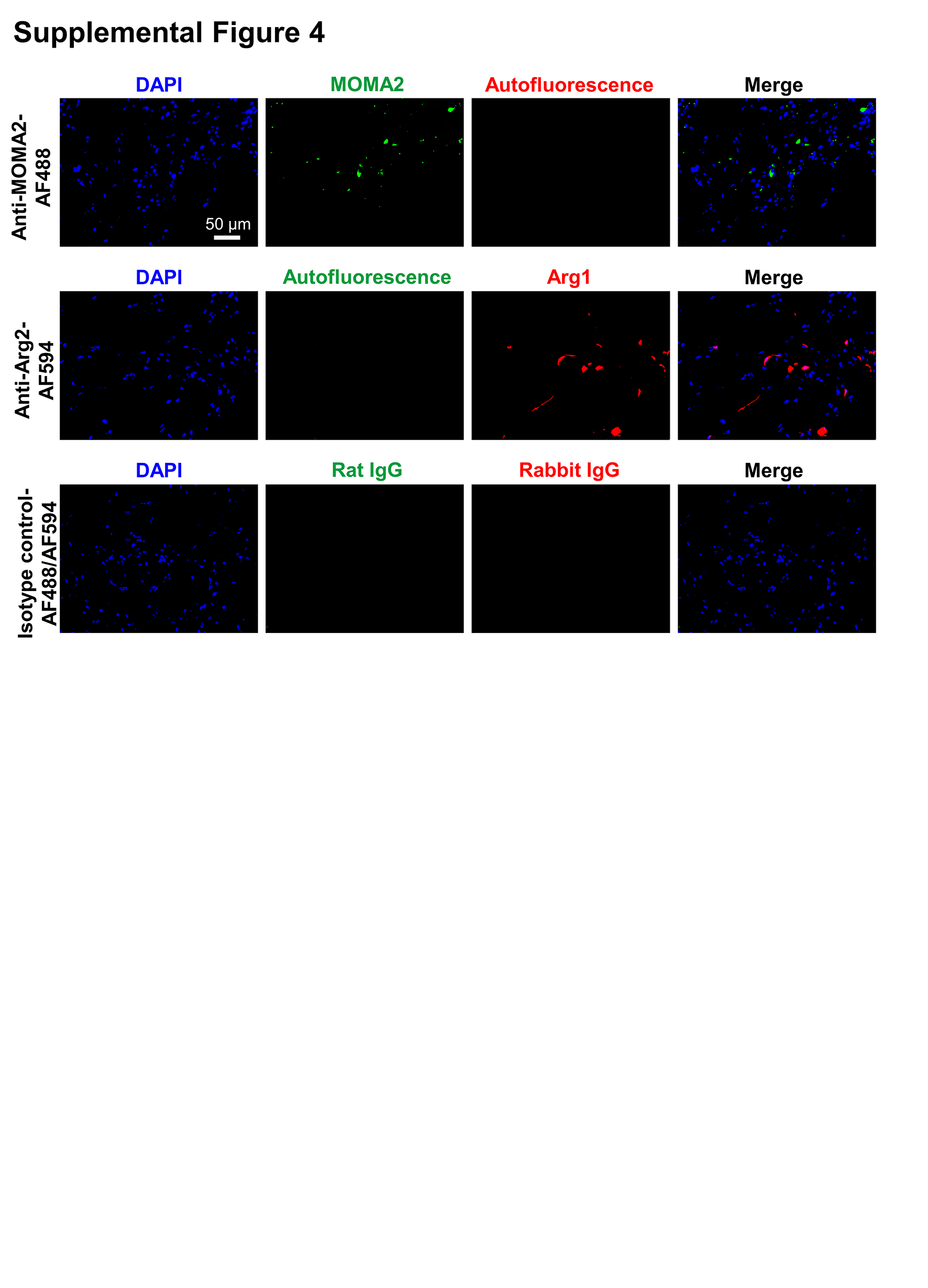

Supplement: Supplementary file 4 [file Image_4.TIF]

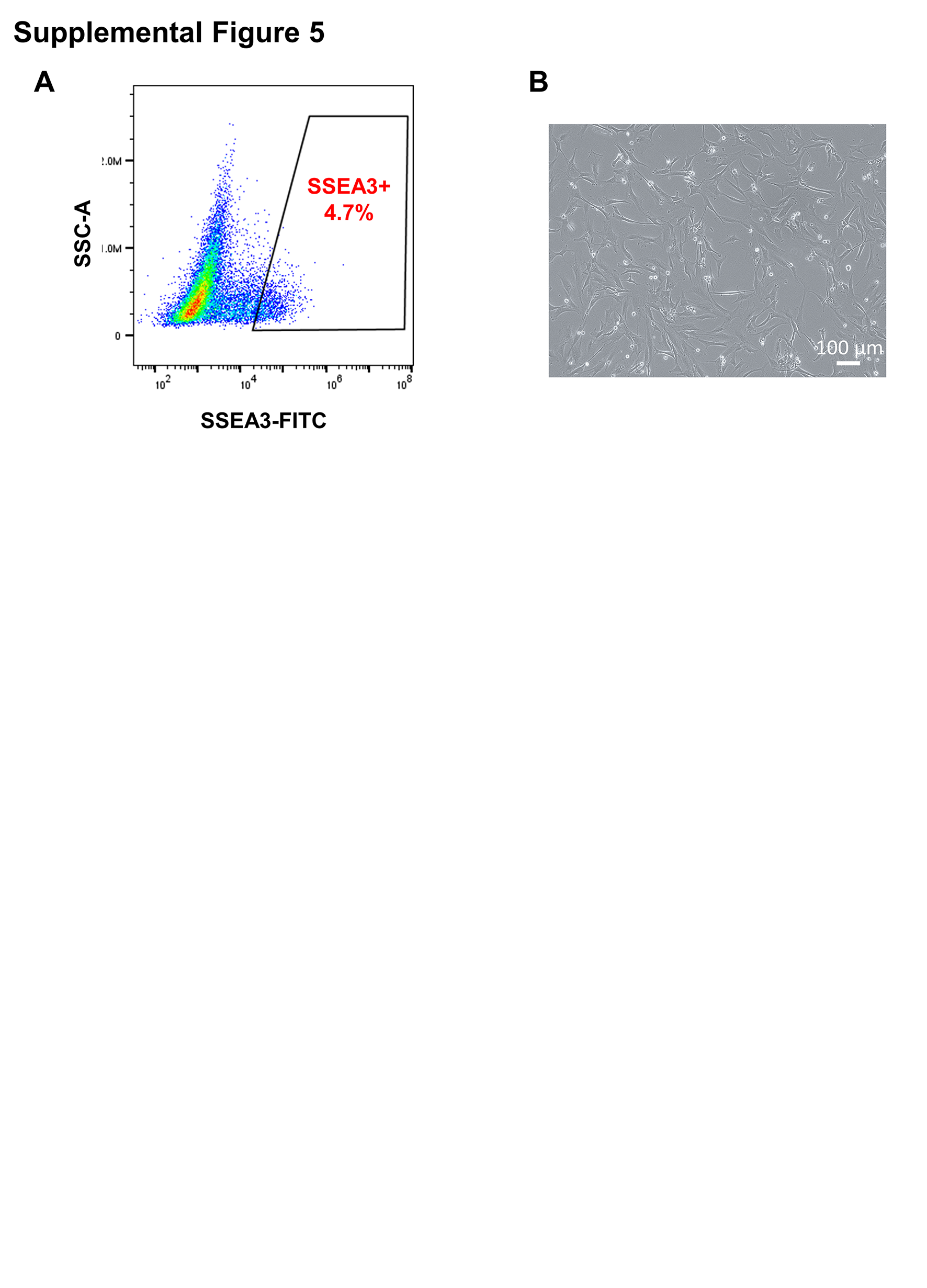

Supplement: Supplementary file 5 [file Image_5.TIF]
